# Integrating multi-platform assembly to recover MAGs from hot spring biofilms: insights into microbial diversity, biofilm formation, and carbohydrate degradation

**DOI:** 10.1186/s40793-024-00572-7

**Published:** 2024-05-06

**Authors:** Kok Jun Liew, Saleha Shahar, Mohd Shahir Shamsir, Nawal Binti Shaharuddin, Chee Hung Liang, Kok-Gan Chan, Stephen Brian Pointing, Rajesh Kumar Sani, Kian Mau Goh

**Affiliations:** 1 Codon Genomics, 42300 Seri Kembangan, Selangor, Malaysia; 2 Faculty of Science, Universiti Teknologi Malaysia, 81310 Skudai, Johor, Malaysia; 3 School of Professional and Continuing Education, Universiti Teknologi Malaysia, 81310 Skudai, Johor, Malaysia; 4 Division of Genetics and Molecular Biology, Institute of Biological Sciences, Faculty of Science, University of Malaya, Kuala Lumpur, Malaysia; 5 Department of Biological Sciences, National University of Singapore, Singapore, Singapore; 6 Department of Chemical and Biological Engineering, South Dakota School of Mines and Technology, Rapid City, SD 57701 USA

**Keywords:** Hybrid assembly, Lignocellulose degradation, Metagenome-assembled genomes, Microbial mat, Thermophile

## Abstract

**Background:**

Hot spring biofilms provide a window into the survival strategies of microbial communities in extreme environments and offer potential for biotechnological applications. This study focused on green and brown biofilms thriving on submerged plant litter within the Sungai Klah hot spring in Malaysia, characterised by temperatures of 58–74 °C. Using Illumina shotgun metagenomics and Nanopore ligation sequencing, we investigated the microbial diversity and functional potential of metagenome-assembled genomes (MAGs) with specific focus on biofilm formation, heat stress response, and carbohydrate catabolism.

**Results:**

Leveraging the power of both Illumina short-reads and Nanopore long-reads, we employed an Illumina-Nanopore hybrid assembly approach to construct MAGs with enhanced quality. The dereplication process, facilitated by the dRep tool, validated the efficiency of the hybrid assembly, yielding MAGs that reflected the intricate microbial diversity of these extreme ecosystems. The comprehensive analysis of these MAGs uncovered intriguing insights into the survival strategies of thermophilic taxa in the hot spring biofilms. Moreover, we examined the plant litter degradation potential within the biofilms, shedding light on the participation of diverse microbial taxa in the breakdown of starch, cellulose, and hemicellulose. We highlight that *Chloroflexota* and *Armatimonadota* MAGs exhibited a wide array of glycosyl hydrolases targeting various carbohydrate substrates, underscoring their metabolic versatility in utilisation of carbohydrates at elevated temperatures.

**Conclusions:**

This study advances understanding of microbial ecology on plant litter under elevated temperature by revealing the functional adaptation of MAGs from hot spring biofilms. In addition, our findings highlight potential for biotechnology application through identification of thermophilic lignocellulose-degrading enzymes. By demonstrating the efficiency of hybrid assembly utilising Illumina-Nanopore reads, we highlight the value of combining multiple sequencing methods for a more thorough exploration of complex microbial communities.

**Supplementary Information:**

The online version contains supplementary material available at 10.1186/s40793-024-00572-7.

## Introduction

Many hot springs in Southeast Asia have been transformed into parks, while only a few still retain their natural surroundings with introduced lignocellulosic plant litters. These sites therefore provide an excellent opportunity to interrogate microbial adaptation to thermophilic utilisation of plant carbohydrate polymers. Research on thermophiles holds potential in biotechnology, especially in industries like bioremediation, biomass conversion, and pulping [1]. Carbohydrate-active enzymes (CAZymes) with inherent thermostability hold great promise for utilising these environments [2].

The isolation of novel thermophiles, a crucial step in unlocking the potential of biomolecules resources, presents significant challenges. Amid these challenges, the metagenome-assembled genome (MAG) approach has emerged as a promising strategy. Currently, most MAG research in the context of hot springs has employed Illumina short-read sequencing, as reflected in various studies [3–9]. To meet the Minimum Information about a Metagenome-Assembled Genome (MIMAG) guidelines, metagenomic bins must exhibit over 80% completeness and less than 5% contamination [10]. Unfortunately, the assembly of short reads often results in fragmented MAGs. The utilisation of long-read sequencing has emerged as an alternative, with platforms like PacBio and Nanopore gaining traction. Kato et al. [11] demonstrated the feasibility of PacBio HiFi long reads for hot spring samples, successfully generating an output of 27.96 Gbp with an N50 of 10,544 bp. They also generated 14 complete and circularised MAGs. Usually, PacBio necessitates a marginally greater quantity and better quality of DNA in comparison to Nanopore. There is currently a scarcity of studies employing long-read Nanopore technology alone, or in combination with Illumina sequencing, for hot spring metagenomic sequencing and MAG assembly.

Several metagenomic studies have reported microbial metabolic adaptation in hot springs based on sequencing data. For example, the distribution and putative role of complete ammonia oxidation (commamox) bacteria in Qinghai-Tibetan Plateau hot springs was revealed [12]. Another study unravelled degradation pathways for lignin-derived aromatic compounds in thermal swamps [13]. In various locations, including tropical hot springs and Yellowstone National Park, amplicon sequencing and shotgun metagenomics revealed the significance of carbohydrate-utilising microorganisms [14, 15]. A study in an Indian hot spring employed functional gene prediction tools (Tax4Fun and Phylogenetic Investigation of Communities by Reconstruction of Unobserved States, PICRUSt) on amplicon data to estimate widespread carbohydrate utilisation across a thermal gradient (43–65 °C) [16]. MAGs and metatranscriptomic data from this site were subsequently analysed [7, 17]. These diverse studies collectively emphasize the universal importance of microbial metabolic adaptation and carbohydrate utilisation in various hot spring environments, providing valuable insights into their ecological roles and potential applications.

In this study, our focus was on the Malaysian Sungai Klah (SKY) geothermal hot spring park, situated in a tropical forest abundant with plant litter and featuring two major types of biofilms [14]. Notably, previously published hot spring metagenomic or MAGs lacked the presence of plant litter in the water. The understanding of the microbiome and its role in adaptation within such ecosystems remains limited. A hybrid assembly strategy combining Illumina and Nanopore reads for MAGs construction was employed in this study. This effort not only provided a comprehensive view of microbial diversity and functional potential but also opened avenues for biotechnological applications, particularly in the realm of carbohydrate degradation within hot spring ecosystems.

## Materials and methods

### Sampling

**Fig. 1:**
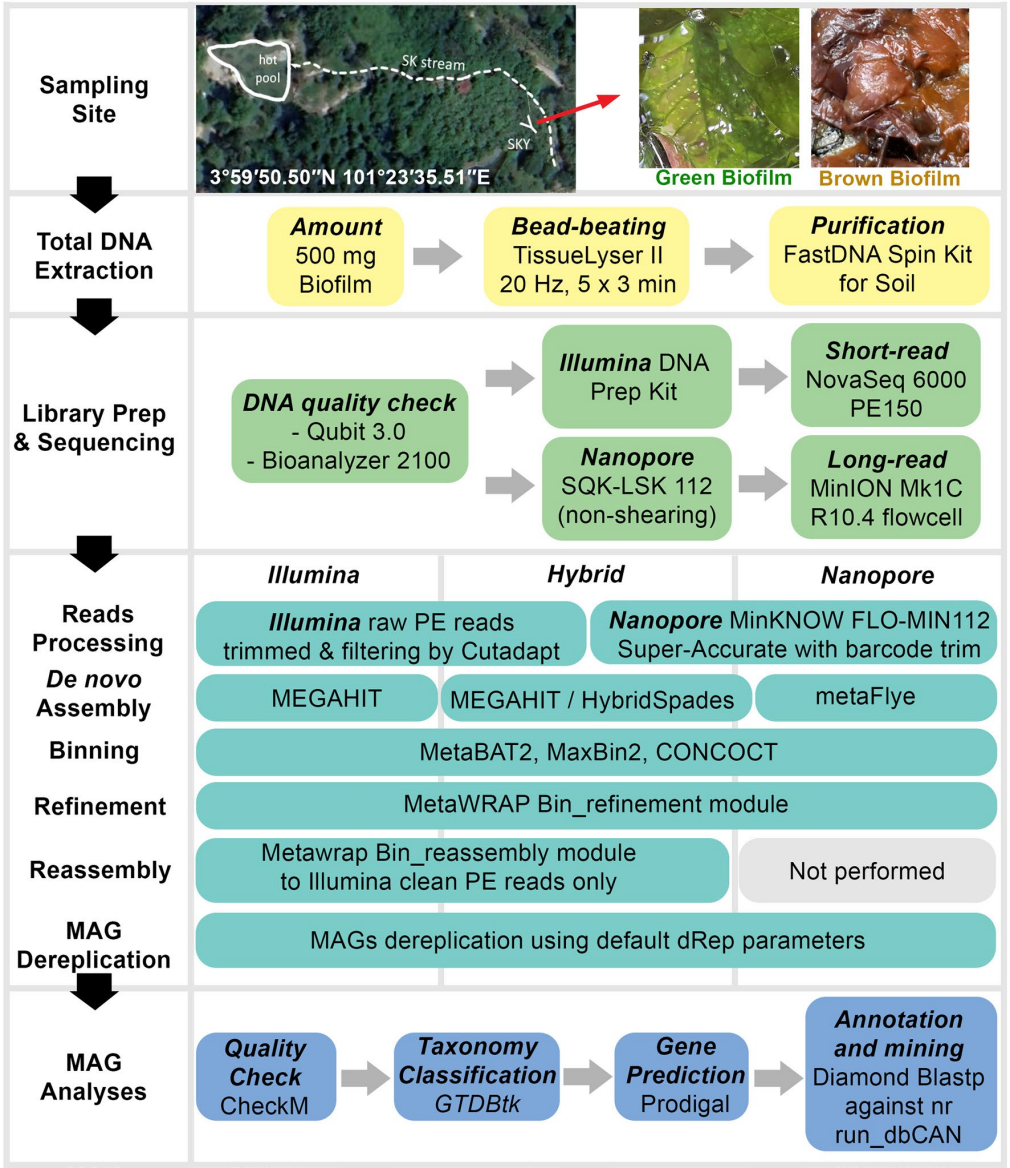
Schematic overview of the study design

The Sungai Klah hot spring is located in Peninsula Malaysia in a tropical rainforest climate (3°59′50.50′′N and 101°23′35.51′′E). The park has a main shallow main stream, featuring temperatures of 60–100 °C and a pH range of 7–9, possesses minimal plant litter and we have previously described microbial diversity in water and sediment of these microhabitats [18]. Alongside the main stream at the SKY site, one encounters submerged leaves and woody plant litter in various stages of decomposition. These include contributions from a diverse array of plant species, such as *Vitex*, *Ficus*, *Stenochlaena*, and *Adenanthera*. The spring head (71–74 °C, pH 8.5) supported brown biofilms whilst green biofilms (58–64 °C, pH 8.5) developed on the surface of the plant litter bed in geothermal waters (Fig. 1) [14, 19]. Sampling was conducted in November 2019 and August 2020 as previously described. Biofilms were recovered and samples preserved at − 20 °C prior to processing. In brief, green biofilms were randomly collected within a half-foot radius into sterile tubes. Approximately 11 feet away from the green biofilm site, we obtained brown biofilm samples in multiple replications. Approximately 500 mg of wet biofilms from each collected sample underwent cell lysis in a TissueLyser II (Qiagen, Hilden, Germany), and genomes were purified using the FastDNA Spin Kit for Soil (MP Biomedicals, Solon, USA). High quality extracted genomes were pooled before sequencing.

### Illumina shotgun metagenome sequencing and MAGs assembly

Our previously sequenced Illumina libraries were used to assemble MAGs as follows [14]. Each of the two green biofilm samples and two brown biofilm samples yielded approximately 20Gb (66.5 million paired-end reads) of data from Illumina NovaSeq 6000 sequencer. We used co-assembly approach for biofilms fastq files. The data (Nov 2019 and Aug 2020) obtained from the green biofilm was concatenated, resulting in approximately 40 Gbp (NCBI SRA Accessions: SRX12118861 and SRX12118862). In the same way, 20Gbp data from each brown biofilm was merged (SRX12118863 and SRX12118864). Trimming and filtering were completed using Cutadapt v3.3 [20] (parameters: -a IlluminaAdapters.fa -A IlluminaAdapters.fa -e 0.1 -O 13 -q 30 –trim-n -m 50) and then de novo assemblies were completed using MEGAHIT v1.2.9 [21] (parameters: –min-count 2 –k-list 21,29,39,59,79,99,119,141). The metaWRAP v1.3 pipeline [22] incorporated MetaBAT2 v2.12.1, CONCOCT v1.0.0, and MaxBin2 v2.2.6 algorithms in the binning process [23–25]. To generate medium- to high-quality MAGs, Bin_refinement (parameters: -c 50—× 10), Blobology, Quant_bins, and Reassembled_bin modules were run within the metaWRAP pipeline. Quality of the MAGs was determined by the CheckM v1.0.12 program [26]. Unless specified, the bioinformatics software was run in default parameters.

### Nanopore sequencing and MAGs assembly

For Nanopore long-read sequencing, both brown and green biofilm genomes underwent one respective flow cell, utilising the Nanopore-suggested protocol SQK-NBD112-24 (Q20 + chemistry on R10.4 flow cell), bypassing genome shearing to obtain long reads. We followed the Nanopore Ligation Sequencing Kit SQK-LSK112 protocol, which included AMPure treatment, adapter ligation, purification, and quantification using Qubit, before loading the samples onto a MinION R10.4 flow cell housed in Mk1C that operated with MinKNOW v.22.05.8. Reads were basecalled using MinKNOW coupled with Guppy v6.1.5, and with basecalling configuration set to FLO-MIN112-Super-Accurate. Barcodes were removed, trimmed, filtered, and reads that passed Q20 (< 5% error) were subjected to de novo assembly by using Flye v2.9 (parameters: –nano-hq –meta). MAGs were constructed using metaWRAP v1.3, with the binning tools MetaBAT2 v2.12.1, CONCOCT v1.0.0, and MaxBin2 v2.2.6. Other modules were carried out as described for the Illumina workflow described above, except that the Reassembled_bin module was not carried out due to the lack of paired-end information in Nanopore reads. Unless specified, the bioinformatics software was run in default parameters.

### Generation of illumina-nanopore hybrid MAGs

To generate hybrid-assembled MAGs, quality control-passed Illumina cleaned reads and Nanopore super-accurate reads were processed either using MEGAHIT or HybridSPAdes. The subsequent construction of MAGs was accomplished using metaWRAP v1.3. Binning was conducted through MetaBAT2 v2.12.1, CONCOCT v1.0.0, and MaxBin2 v2.2.6. Other modules in metaWRAP were carried out as usual, except that the Reassemble_Bins modules were performed using only the Illumina reads.

### MAG dereplication and data deposition

To compare and evaluate the MAGs generated from Illumina, Nanopore, and the hybrid assembly of both, we employed the dRep program (parameters: -l 50,000 -comp 75 -con 25 –checkM_method lineage_wf –S_algorithm fastANI –n_PRESET normal -pa 0.9 -sa 0.95 -nc 0.1 -cm larger –clusterAlg average -comW 1 -conW 5 -strW 1 -N50W 0.5 -sizeW 0 -centW 1) [27]. Genome Database Taxonomy (GTDB-Tk v.1.7.0) was used for taxonomy annotation for all MAGs [28]. Only dereplicated MAGs were deposited in NCBI Genbank database. The constructed MAGs were linked with the respective BioSample accession numbers of SAMN37333998–SAMN37334222. Each of the MAGs was also deposited as Whole Genome Sequence (WGS) in Genbank database with accession numbers of JAVQVT000000000–JAVQZZ000000000 (dereplicated MAGs from green biofilms) and JAVQRJ000000000–JAVQVS000000000 (dereplicated MAGs from brown biofilms). In addition, the MAGs generated in this study are also accessible through the NCBI BioProject PRJNA1012858. Please refer to Additional file 1: Table S1 for more information.

### Phylogenomic analysis of MAGs

GToTree v1.8.1 was used to analyse the phylogenomics of all MAGs (parameters: -H Bacteria_and_Archaea -D -T IQ-TREE). A total of 25 marker genes of bacterial and archaeal was used in the analyses. The output tree files (.tre) were visualised by iTOL v6.8 with proper colouring and annotations of the MAGs based on the taxonomy as identified by GTDB-Tk.

### Functional annotation of MAGs

Prodigal v2.6.3 was utilised to identify open reading frames (ORFs). Protein sequences related to heat shock proteins were matched against the heat shock protein information resource (HSPIR) database [29]. Proteins involved in carbohydrate utilisation were identified via the Carbohydrate-Active EnZymes (CAZy) database using run_dbCAN v3.0 [30], and the selected sequences needed to demonstrate positivity in at least one of three tests using HMMER, Hotpep, or Diamond. Sequences of ABC-type sugar transporters, major facilitator superfamily (MFS), sodium solute symporters, and the phosphotransferase system were retrieved from InterPro or UniProtKB. If required, sequence verification was conducted using Diamond v2.0.14, BlastP searched against the NCBI non-redundant (nr), SwissProt, InterPro, and Protein Data Bank (PDB) databases.

## Results

### Site description

SKY hot spring is a unique high-temperature spring known to be filled with plant litters. In a previous publication, we investigated prokaryotic and eukaryotic diversity in two biofilms using 16S and 18S rRNA amplicon sequencing. For a better understanding of the sampling site and the bioinformatics protocol used in this study, please refer to Fig. 1 and our earlier report [14]. The SKY hot spring, with a relatively small water body, experiences limited water movement and exhibits a temperature range of 58–64 °C, while the spring head maintains an average temperature of 71–74 °C, and the average pH value remains around 8.5. The water chemistry analysis revealed the following concentrations: total organic carbon (TOC) at 0.8 mg/L, total nitrogen at 1.8 mg/L, sulfur at 2.7 mg/L, sulphate at 5 mg/L, and bicarbonate at 27 mg/L. Other hot springs in Malaysia that lack plant litter often have TOC values ranging from 0 to 0.4 mg/L.

Based on amplicon data, the green biofilm exhibited dominance with approximately 50–60% *Cyanobacteria*. Together with *Bacteroidota* and *Chloroflexota*, these three phyla constituted nearly 90% of the total detected amplicon sequence variants (ASVs) [14]. In the brown biofilm, *Chloroflexota* dominated half of the ASVs, while major ASVs from *Bacteroidota*, *Thermotogota*, and *Armatimonadota* collectively constituted about 20–40% of the community composition. A diverse presence of other bacterial phyla, each exceeding 1% abundance, was also noted. *Crenarachaeota* was the sole major archaeal phylum observed in both samples.

### Comparison of illumina-based MAGs, nanopore-based MAGs, and illumina + nanopore hybrid MAGs

We performed shotgun sequencing of the two biofilms collected in Nov 2019 and Aug 2020, with each biofilm subjected to two runs on Illumina NovaSeq, generating approximately 20 Gbp output reads per run from short reads. Assembly was performed using MEGAHIT. Employing the default metaWRAP pipeline setting, each shotgun sequencing run yielded an average of 70 MAGs per dataset for each sampling (data not shown). Due to the similarity of microbiota types between the 2019 and 2020 samples and the high redundancy between MAGs, we decided to employ a co-assembly strategy. This involved pooling fastq files of the same biofilm type (i.e., 20 Gbp + 20 Gbp raw data from green biofilm or brown biofilm) before running the MEGAHIT and metaWRAP pipeline simulations. This strategy allowed us to generate a higher number of MAGs, resulting in 132 medium to high quality MAGs for the green biofilm and 131 medium to high quality MAGs for the brown biofilm (Table 1).

**Table 1:** Overall statistics and quality of MAGs assembled in each biofilm type using Illumina-reads, Nanopore-reads, and Illumina + Nanopore hybrid reads

Biofilm type	Green biofilm sample (58–64 °C)				Brown biofilm sample (71–74 °C)			
Sampling date	2019 + 2020	2020	2019 + 2020	2019 + 2020	2019 + 2020	2020	2019 + 2020	2019 + 2020
Sequencer	Illumina NovaSeq	Nanopore R10.4 K12	Illumina + Nanopore	Illumina + Nanopore	Illumina NovaSeq	Nanopore R10.4 K12	Illumina + Nanopore	Illumina + Nanopore
Reads assembler	MEGAHIT	Fyle	MEGAHIT	HybridSPAdes	MEGAHIT	Fyle	MEGAHIT	HybridSPAdes
**Overall statistics**								
High-quality MAG (numbers)	70	11	69	76	58	5	62	73
Medium-quality MAG (numbers)	62	18	73	65	73	11	77	62
Total MAGs	132	29	142	141	131	16	139	135
Domain archaea	4	0	4	3	42	3	45	44
Domain bacteria	128	29	138	138	89	13	94	91
Average GC %	55	60	55	55	49	56	49	49
Min. % completeness	52.3	53.8	50.8	50.5	50.8	50.2	51.0	50.1
Max. % completeness	100	99.27	100	100	100	98.7	100	100
Average % completeness	85.4	84.0	83.4	85.4	84.9	79.7	84.1	86.1
Median % completeness	91.3	87.6	89.2	91.5	89.5	85.9	89.4	92.4
Min. % contamination	0	0	0	0	0	0	0	0
Max. % contamination	8.5	7.7	8.7	8.2	8.3	9.3	9.2	9.7
Average % contamination	1.4	2.0	1.6	1.4	1.4	3.2	1.6	1.2
Median % contamination	1.1	1.7	1.2	1.1	0.9	2.6	1.0	0.6
Min. N50	1,899	53,975	2,197	1,858	2,073	49,744	1,690	1,579
Max. N50	422,056	3,806,896	340,768	775,088	733,637	3,082,426	1,112,774	1,408,406
Average N50	58,550	1,010,611	48,584	77,279	83,520	737,298	82,979	109,964
Median N50	23,183	480,052	19,069	27,933	39,712	413,089	33,081	45,706
**Contigs statistics**								
Least fragmentised MAG (contig numbers in that MAG)	9	1	11	6	6	1	3	4
Most fragmentised MAG (contig numbers in that MAG)	1,286	131	1,387	1,320	1,249	86	904	1,131
Average number of contigs	309	32	315	298	179	19	178	164
Median number of contigs	219	21	217	173	100	13	112	95
Min. contig size (bp)	401,200	1,749,479	348,994	441,126	451,946	990,151	489,583	387,085
Max. contig size (bp)	5,447,934	6,970,065	5,389,216	5,442,895	6,071,299	5,189,004	6,058,125	6,065,741
Average contig size of all MAGs (bp)	2,752,890	3,069,724	2,703,622	2,751,768	1,821,238	2,277,883	1,792,544	1,857,170
Median contig size of all MAGs (bp)	2,685,960	2,938,892	2,769,042	2,685,789	1,614,897	2,024,692	1,595,886	1,657,254

Utilising the Nanopore R10.4 flow cells, we sequenced genomes extracted from green and brown biofilms collected in August 2020. Although the output reads were lower compared to the R9 series, the R10.4 flow cell with K12 chemistry demonstrated improved accuracy [31]. We obtained approximately 5.2 Gbp of high-accurate long reads for the green biofilm and 3.6 Gbp for the brown; these long reads were subsequently assembled using Flye. Despite the lower output, we successfully obtained 29 MAGs for the green sample and 16 for the brown biofilm (Table 1). It is worth noting that the average (and median) completeness of MAGs generated by Nanopore is lower than that of Illumina-MAGs. Additionally, the estimated contamination in Nanopore-generated MAGs is relatively high on average (and median) compare to Illumina-MAGs.

To explore the potential benefits of combining Illumina and Nanopore sequencing data, we conducted a hybrid assembly approach for each green and brown biofilm, utilising Illumina reads (20 + 20 Gbp) and Nanopore reads, respectively. The assembly process was performed using MEGAHIT or HybridSPAdes. The summary of key statistics for the obtained MAGs is presented in Table 1. Comparing the performance of the hybrid assemblers, MEGAHIT and HybridSPAdes, both generated a higher number of total MAGs compared to using Illumina reads alone. In terms of MAG completeness, HybridSPAdes exhibited better performance than MEGAHIT for our dataset. On average, the hybrid assemblers demonstrated an improvement in N50 when compared to Illumina-MAGs. Additionally, hybrid-MAGs showcased reduced total numbers of contigs, indicating a less fragmented genome, although this improvement was not consistent throughout the entire dataset.

### Taxonomy of dereplicated MAGs

To compare and evaluate the MAGs generated from Illumina, Nanopore, and the hybrid assembly of both, we employed the dRep program. Our goal was to select the highest quality MAGs specific to each biofilm sample, aiding in the identification of reliable and representative genomes for our downstream analysis. The total numbers of selected MAGs were summarised in Table 2.

**Table 2:** Summary of dereplicated MAGs

Sample type	Illumina-MAGs (MEGAHIT)	Nanopore-MAGs (Flye)	Hybrid-MAGs (MEGAHIT)	Hybrid-MAGs (HybridSPAdes)	Total dereplicated MAGs
Green biofilm	22	6	25	58	111
Brown biofilm	26	2	28	58	114

Dereplicated MAGs were obtained separately for the green and brown biofilm data sets. However, dereplication between the green and brown biofilms was not carried out. Many Nanopore-derived MAGs were not chosen by dRep probably because the quality of Nanopore-derived MAGs may not be on par with Illumina-derived MAGs or hybrid MAGs generated from both Illumina and Nanopore. Due to the likelihood that the depth is not sufficient, the Nanopore-derived MAGs for this work have slightly lower completeness and higher contaminants compared to the counterparts in pure Illumina- or hybrid-MAGs. Despite this, hybridising short and long reads has improved the overall quality metrics; hence, most of dRep-selected MAGs were from hybrid techniques. In other words, Nanopore sequencing is still essential in this work, because it further enhance the overall quality of MAGs.

**Fig. 2:**
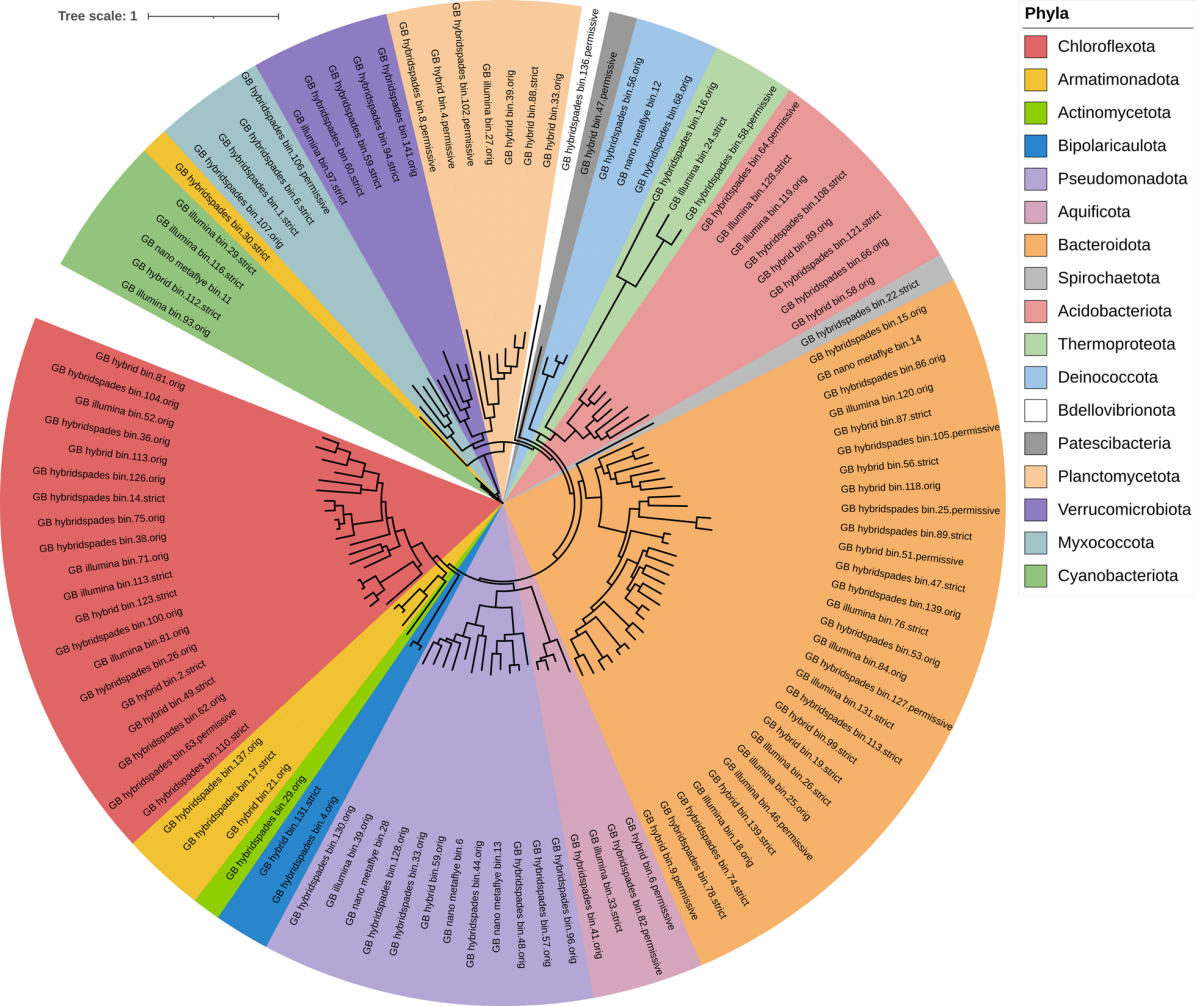
Maximum likelihood phylogenetic tree of MAGs (green biofilms, GB) based on the alignment of 25 marker genes in GToTree and visualised by iTOL

The majority of MAGs (total 108) in the green biofilm sample were found to be associated with the Bacteria domain and fell within 17 phyla. Phyla with highest numbers of MAGs included *Acidobacteriota* with eight MAGs, *Bacteroidota* with 29 MAGs, *Chloroflexota* with 20 MAGs, *Proteobacteria* with 13 MAGs, *Planctomycetota* with seven MAGs, and *Verrucomicrobiota* with five MAGs. Additionally, several minority phyla were present in the sample, including *Actinobacteriota*, *Bdellovibrionota*, *Bipolaricaulota*, *Deinococcota*, *Myxococcota*, *Patescibacteria*, *Spirochaetota*, and others (Fig. 2, Additional file 1: Table S1). We also constructed three MAGs within the Archaea domain, with all three falling under the phylum *Thermoproteota*.

The MAGs obtained from the green biofilm sample contained five Cyanobacterial taxa, including species within the genera *Gloeomargarita* and *Geminocystis* that are novel records for hot springs. Both MAGs are classified at the identical class level (*Cyanobacteriia*) and order level (*Cyanobacteriales*), indicating a potential ecological connection. *Gloeomargarita* is known for its ability to form multicellular filaments, while *Geminocystis* spp. are solitary, spherical, or slightly oval and non-filamentous [32]. The other three MAGs could only be classified at the family level: *Pseudanabaenaceae*, *Oscillatoriaceae*, and *Neosynechococcaceae*.

A significant number of MAGs in both biofilm datasets remained poorly phylogenetically classified due to limited available taxonomy information. For example, in the case of the green biofilm sample, out of the total 111 dereplicated MAGs, nearly 50% of them cannot be accurately assigned to a specific family based on the nomenclature of cultured type strain representatives. These MAGs are characterised by a lack of taxonomic resolution, with few of them only able to be classified at the order level. Furthermore, few MAGs from the green biofilm dataset could not even be assigned to the class level (HRBIN16 and UBA11346), with only their phylum information known (*Armatimonadota* and *Planctomycetota*).

**Fig. 3:**
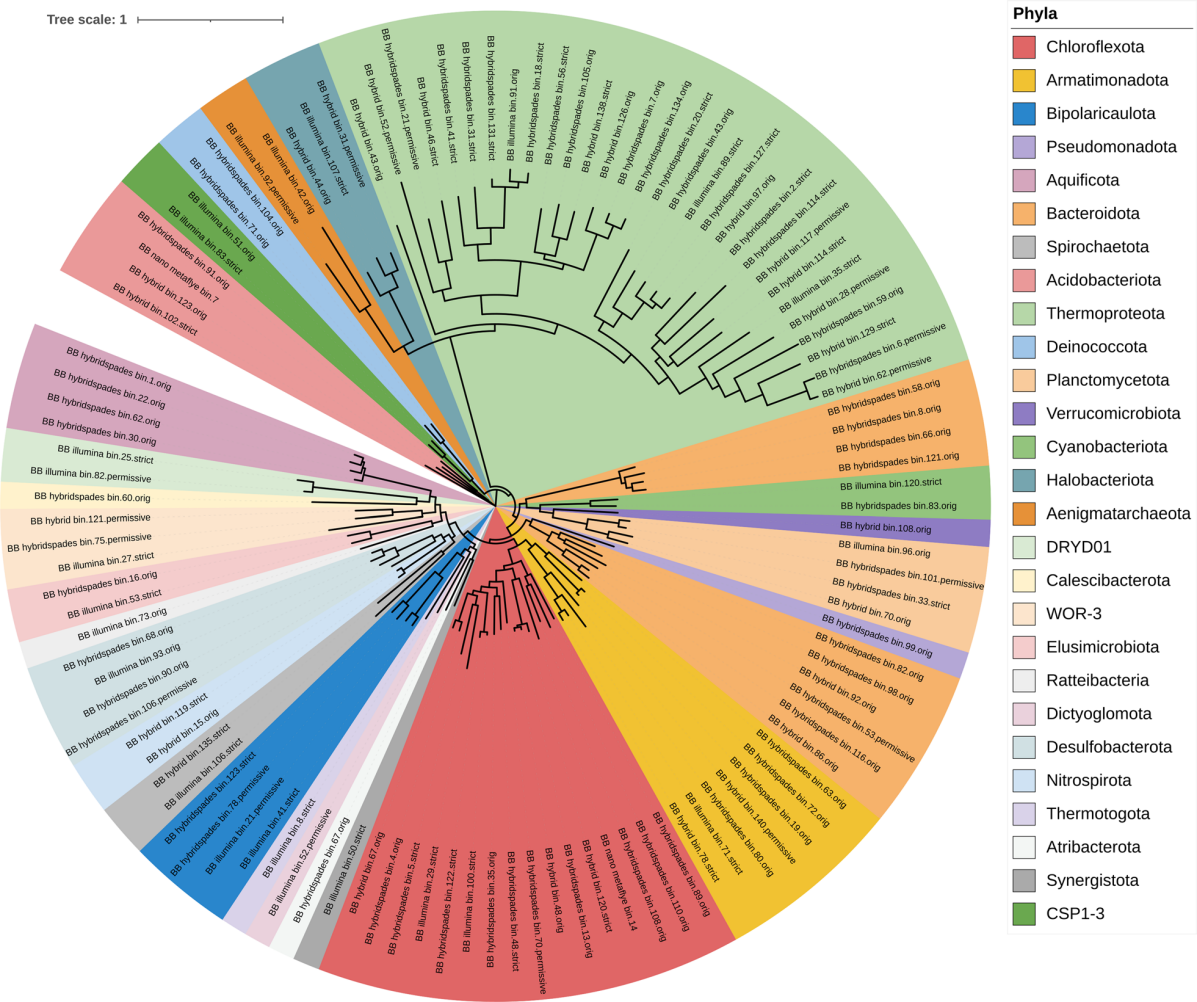
Maximum likelihood phylogenetic tree of MAGs (brown biofilms, BB) based on the alignment of 25 marker genes in GToTree and visualised by iTOL

**Fig. 4:**
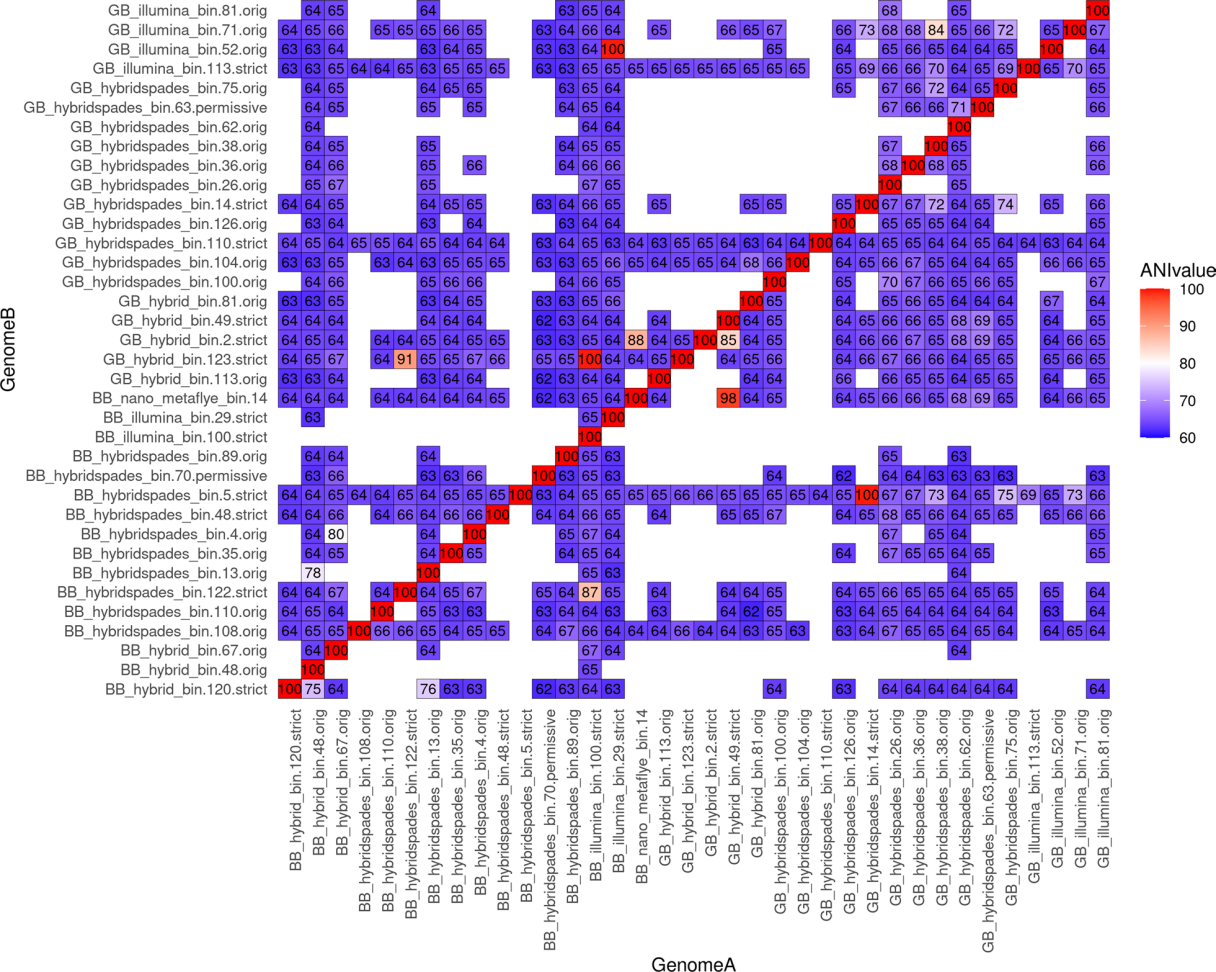
Heat map displaying ANI comparison between *Chloroflexota* MAGs in green- and brown biofilm

The brown biofilm, located near the hot spring head with significantly higher temperatures compared to the green biofilm, was subjected to MAG taxonomic analysis (Fig. 3, Additional file 1: Table S1). The detected archaea MAGs in brown biofilm were higher in numbers and more diverse than in green biofilm. We detected Archaea from three distinct phyla: *Aenigmatarchaeota*, *Halobacteriota*, and *Thermoproteota*, with MAG counts of 2, 4, and 30, respectively. The few *Thermoproteota* MAGs were extremely likely to be species from the genera Candidatus *Caldarchaeum*, Candidatus *Korarchaeum*, Candidatus *Nitrosocaldus*, Candidatus *Caldarchaeum*, and *Ignisphaera*, whilst many other MAGs could only be placed at a higher level within the *Thermoproteota*. In this current work, 16 MAGs related to *Chloroflexota* were constructed. Figure 4 summarises the average nucleotide index (ANI) between the two biofilms that were identified as *Chloroflexota* MAGs.

### Energy metabolisms in biofilms

Since the taxonomy of the community in the biofilm is complex, we expected to encounter taxa that syntrophically support each other in obtaining nutrients. Green biofilm was dominated primarily by aerobic and anaerobic photoautotrophic *Cyanobacteria* that derive their energy from light and CO_2_. In the green biofilm, we identified multiple *Chloroflexota* MAGs, one of which is likely affiliated with the *Chloroflexus* genus. However, this MAG does not match any known species or subspecies of *Chloroflexus aggregans* [33] due to low ANI similarity. Another dominant MAG in the green biofilm was identified as *Bacteroidota*. We constructed 29 *Bacteroidota* MAGs, with only one confidently classified as *Ignavibacterium*. This genus comprises non-photrophic heterotrophs, suggesting that many other MAGs related to *Bacteroidota* survive by using organic compounds as energy and carbon sources through chemoheterotrophy. Another main phylum in green biofilm was *Acidobacteriota*. Most members of this phylum are probably organotrophs and use chemoautotrophy for energy production. One of the MAGs in the green biofilm has a high ANI with genus *Chloracidobacterium*. It is worth noting that *Chloracidobacterium thermophilum*, a type strain, is the only chlorophyll (Chl)-dependent phototrophic genus in the *Acidobacteria* phylum [34]. In short, in the green biofilm, the main energy sources are derived from light and CO_2_, with aerobic and anaerobic photoautotrophic *Cyanobacteria* dominating.

The types of phyla for MAGs constructed for the brown biofilm dataset are summarised in Fig. 3. *Chloroflexota* is known for its ability to perform anoxygenic phototrophy and aerobic respiration. *Aquificaceae*, *Kapabacteriales*, *Kryptoniaceae*, and *Armatimonadota* inhabit the brown biofilm and are likely to rely on chemoorganotrophic, chemoheterotrophic, or oligotrophic metabolism for their thriving. Furthermore, certain *Thermoproteota* MAGs belong to heterotrophic species within the genera Candidatus *Caldarchaeum* and Candidatus *Korarchaeum*. Additionally, Candidatus *Nitrosocaldus* is known for its autotrophic and chemolithoautotrophic characteristics, Candidatus *Caldarchaeum* is probably chemoorganotrophic or chemoheterotrophic, and *Ignisphaera* typically exhibits chemoorganotrophic traits [35]. In addition, *Pseudothermotoga*, another bacterium found in brown biofilm, is thermophilic, anaerobic, fermentative, and hydrogen-producing. Collectively for the brown biofilm, energy sources vary across different phyla.

### Macromolecules involved in biofilm formation

Based on genome annotation, Cyanobacterial MAGs detected in the green biofilms contained genes associated with various aspects of type IV pilus biogenesis, twitching motility, and bacterial adhesion in Cyanobacteria, including leader peptidase (prepilin peptidase), pilus biogenesis proteins (PilF, PilM, PilQ), fimbrial assembly ATPase (PilB), type IV pilin (PilA), fimbrial assembly protein (PilC), and twitching motility protein (PilT/ PilU family). Pili or fimbriae are short and thin non-flagellar appendages that facilitate Cyanobacterial adherence to surfaces [36]. Additionally, these MAGs showed a noticeable presence of proteins associated with the LuxR family, a two-component transcriptional response regulator. This suggests a potential role in signal transduction and gene regulation, particularly in processes such as quorum sensing.

We identified proteins associated with exopolysaccharide biosynthesis in MAGs related to *Cyanobacteria*, including the polyprenyl glycosylphosphotransferase (Wzx or Flippase). This exopolysaccharide biosynthesis enzyme plays a crucial role in translocating the repeating units of exopolysaccharides across the inner membrane of bacteria. Interestingly, the MAG related to *Neosynechococcaceae* exhibited an additional type of polysaccharide biosynthesis specific to hormogonium polysaccharide. In addition to exopolysaccharide biosynthesis, our analysis revealed the presence of protein sequences related to polysaccharide deacetylases, lipopolysaccharide export systems, polysaccharide export proteins, capsular polysaccharide biosynthesis, and nucleoside-diphosphate-sugar pyrophosphorylase [37].

Besides *Cyanobacteria*, it is likely that members of the phylum *Chloroflexota*, represented by MAGs, also contribute to the formation of green biofilm matrices. Within the total *Chloroflexota* MAGs identified in the green biofilm, six of them showed high similarity in ANI values to the genera *Chloroflexus*, *Caldilinea*, or Candidatus *Roseilinea*. These genera are known to form filamentous biofilms [33]. Genomic analysis of several *Chloroflexota* MAGs revealed the presence of related genes or proteins associated with pilus assembly, such as general pilus assembly proteins, Flp pilus assembly complex ATPase component TadA, Flp pilus assembly protein CpaB, RcpC, and CpaF, Flp family type IVb pilin, prepilin peptidase, among others.

The physical appearance of the brown biofilm suggested greater biocomplexity. It consists of a combination of slimy, intertwined with thin, elastic, jelly-like reddish-brown biofilm. The reddish-brown hue is likely caused by *Roseiflexus*-like MAGs that belongs to phylum *Chloroflexota*. These MAGs likely contribute to EPS formation through the action of proteins such as polysaccharide biosynthesis protein, polysaccharide biosynthesis C-terminal domain-containing protein, and polysaccharide deacetylase family protein. While the taxonomy of *Chloroflexota* in the brown biofilm differs from the green biofilm, the overall principle of biofilm formation is expected to be similar.

Occasionally, light grey or whitish fibrous biofilm is also present alongside the brown biofilm, possible linked to four *Aquificaceae*-like MAGs. This fibrous biofilm resembles the one documented in thermal streams in Russia [38]. In the constructed MAGs, we identified Type IV twitching motility protein PilT, PilT/PilU family pilus ATPase, pilus assembly protein PilM (closely related to *Hydrogenobacter* type), pilus assembly protein, and pre-pilin peptidase. Additionally, the phylum *Thermotogota* stands out as one of the prominent taxonomic groups identified in the brown biofilm sample, and *Thermotoga maritima* is known for its capacity to produce exopolysaccharides [39].

### Heat stress adaptations

We have visited SKY hot spring multiple times, and we observed fluctuations in water temperature with variations of 3–5 °C. Based on these observations, we anticipate that molecular chaperones play a crucial role in maintaining protein homeostasis and mitigating the impact of protein denaturation and proteotoxicity caused by sudden temperature changes.

**Fig. 5:**
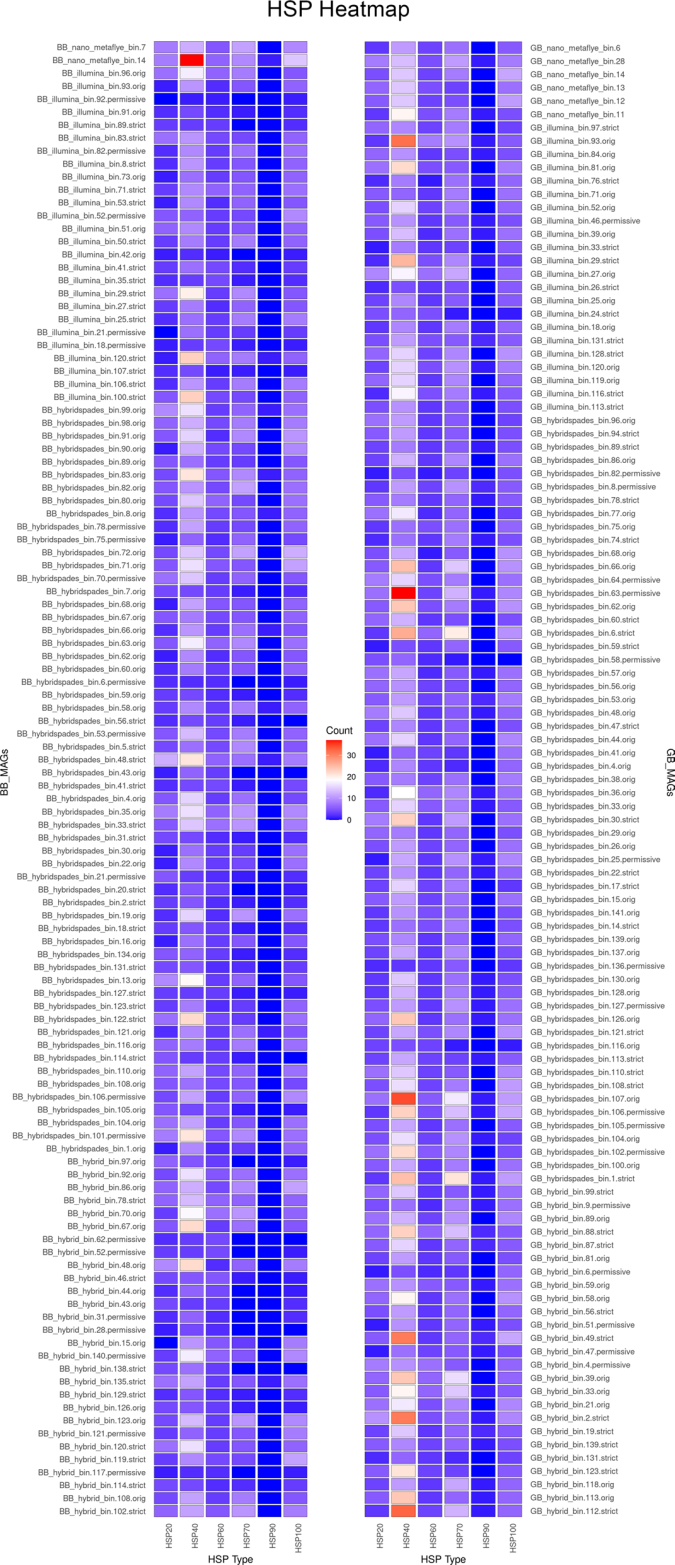
Heat map displaying HSP20, HSP40, HSP60, HSP90, and HSP100

To protect their functional proteins, thermophiles employ various strategies including heat shock proteins (HSPs), chaperones, chaperonins, and α- and β-subunit prefoldins [40]. In our study, we conducted protein sequence search for HSP20, HSP40, HSP60, HSP90, and HSP100 in all the MAGs and visualised them in a heat map (Fig. 5). In general, more HSP40 sequences were detected, while HSP90 were found to be least abundant. HSP40, also denoted as DNAJ or DnaJ/Hsp40 homologs, is primarily involved in assisting protein folding, preventing protein aggregation, and maintaining the integrity of protein quality control. HSP60, known as chaperonins (chaperonin GroEL), is crucial for proper protein folding and preventing aggregation. HSP90 proteins (HtpG) are indispensable for protein maturation and stabilisation. HSP100 proteins, including ClpB, belong to an ATP-dependent chaperone family and aid in the disaggregation and reactivation of denatured or aggregated proteins caused by stress conditions.

### Starch and lignocellulosic degradation and sugar transporter

**Fig. 6:**
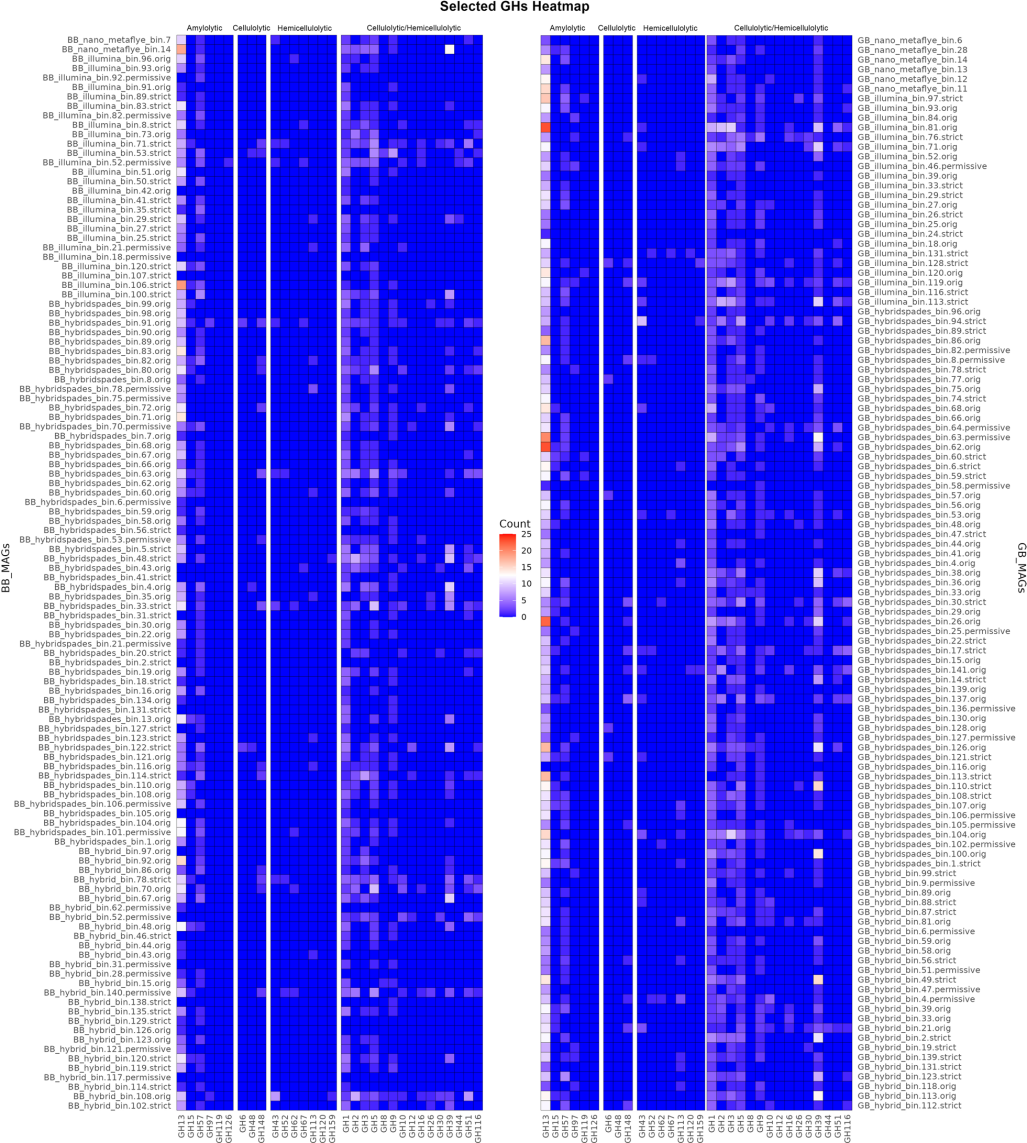
Heat map displaying CAZymes from selected MAGs

**Fig. 7:**
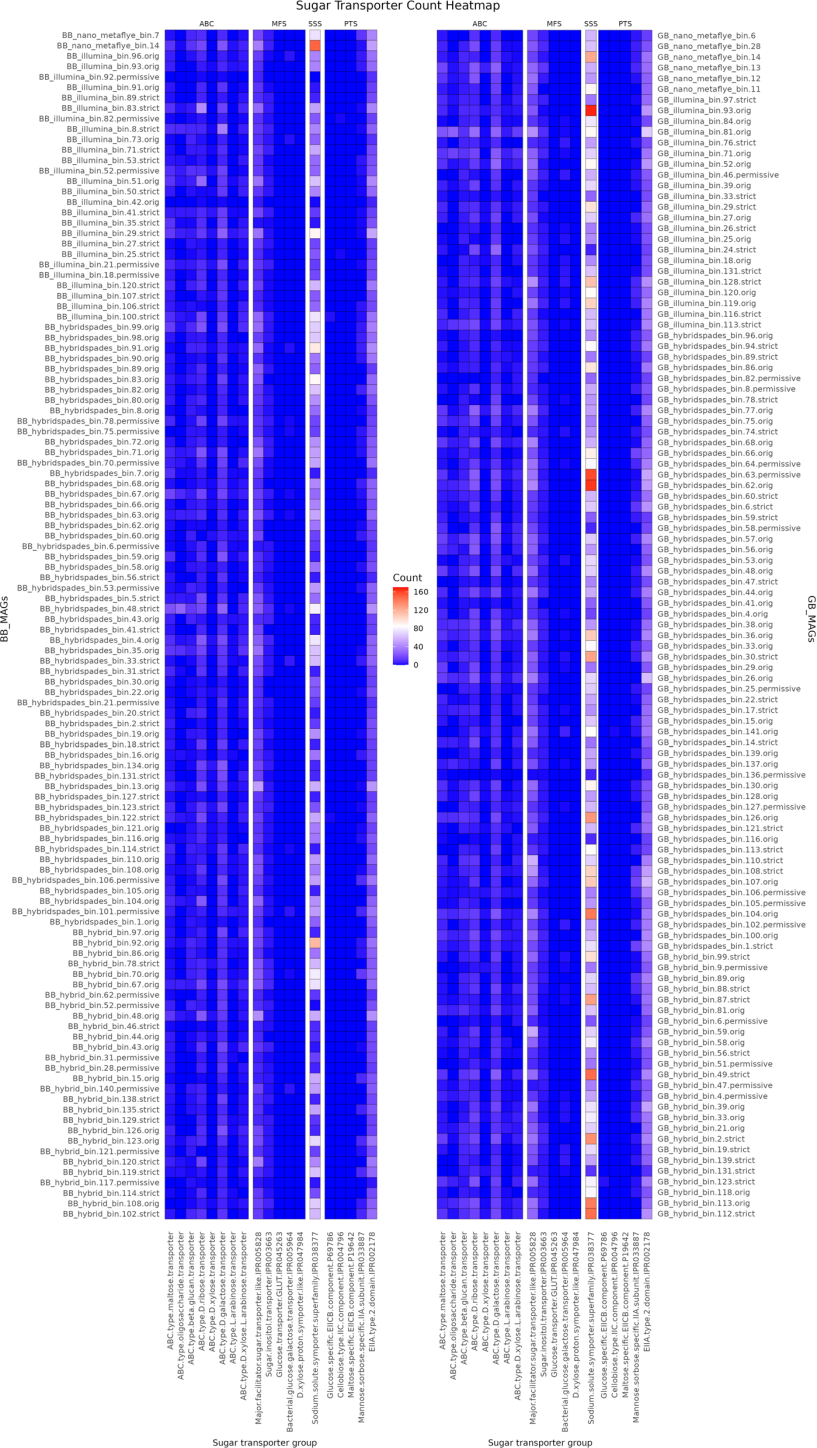
Heat map displaying sugar transporters in selected MAGs arranged according to ABC type transporter (ABC), major facilitator superfamily (MFS), solute/sodium symporter (SSS), and phosphotransferase system (PTS) transporter

An overview of the putative CAZymes in all the dereplicated MAGs is shown in Fig. 6. The proteins were grouped according to the main catalytic reactions, i.e., amylolytic enzymes, cellulolytic enzymes, and hemicellulosic enzymes. Since certain glycosyl hydrolases (GH) groups (i.e., GH1, GH2, GH3, GH5, etc.) consist of a mixture of cellulolytic and hemicellulosic enzymes, we have therefore separated them in the heatmap. Extracellularly expressed hydrolases will cleave the carbohydrate polymers, and eventually, a broad range of sugar transporters (Fig. 7) will import these monomeric, dimeric, or short polymerisation degree sugar chains for energy consumption and other biochemical pathways.

Cyanobacterial MAGs displayed sequences that were potentially involved in starch or glycogen metabolism. We only identified α-mannosidase, β-glucosidase (GH1, GH3, and GH116), and endo-1,4-β-xylanase among the enzymes related to cellulosic hydrolysis. Notably, we did not find several key enzymes essential for cellulase, endoglucanase, cellodextrinase, α-glucuronidase, β-xylosidase, reducing-end-xylose releasing exo-oligoxylanase, and arabinan active enzymes.

*Chloroflexota* MAGs may express a wide range of hydrolases, each of which performs a specialised function in the breakdown of complex polysaccharides within or adjacent to the biofilm matrix. These putative enzymes are involved in the hydrolysis of starch, pullulan, or glycogen, including α-amylase, trehalose synthase, glycosyl hydrolases and pullulanase, among others. Another set of enzymes target xylan and cellulose, including 1,4-β-xylosidase, endo-1,4-β-xylanase, and β-glucosidase. Arabinogalactan β-L-arabinofuranosidase, endo-1,4-β-galactanase and L-arabinose isomerase participate in the hydrolysis of arabinogalactans, while β-mannosidase and α-mannosidase act on mannose-containing compounds. Collectively, these diverse hydrolases play a crucial role in breaking down complex carbohydrates within or adjacent to the biofilm matrix, highlighting the remarkable metabolic versatility of *Chloroflexota* MAGs in high temperature plant litter decomposition.

Within our dataset, we discovered representatives from the class *Armatimonadota* present in the MAGs related to HRBIN16 and HRBIN17, along with additional classes like *Abditibacteria* and *Fimbriimonadia*. Notably, our analysis unveiled a diverse array of glycosyl hydrolase sequences associated with various MAGs within the *Armatimonadota* phylum. These putative enzymes include endoglucanases, exoglucanases, β-glucosidases, xylanases, mannanases, arabinofuranosidases, acetyl xylan esterases, α-glucuronidases, and β-xylosidases.

In the context of a high-temperature hot spring, it becomes evident that the microbiota engages in a symbiotic decomposition of plant litter. Take, for instance, a MAG affiliated with *Bipolaricaulaceae*, which features five distinct intracellular β-glucosidases (devoid of signal peptides) but lacks other hydrolases such as cellulose. While other members of the biofilm kickstart the degradation of cellulosic materials, this bacterium *Bipolaricaulaceae* strategically employs its β-glucosidases to break down short oligosaccharides. This enzymatic action converts the oligosaccharides into glucose, serving as an energy source for the bacterium.

Expanding our investigation, we explored the vast landscape of CAZymes, finding a plethora of novel enzymes in diverse taxa. Table 3 shows some examples of enzymes, namely endoglucanases, xylanases, β-xylosidases, and β-glucosidases, for their sequence similarities with the closest counterparts.

**Table 3:** Selected putative cellulase, endoglucanase, xylanase, β-glucosidase and β-xylosidase from the selected MAGs and the closest hits. Refer to Additional file 2: Table S2 for the protein sequences

GH	MAG’s phylum	Closest identity (%) Blastp RefSeq	Closest identity (%) PDB (PDB ID)
**Cellulase/endoglucanase**			
5	*Planctomycetota*	60% Candidatus *Fervidibacter sacchari*	49% *Thermotoga maritima* (7EC9)
5	*Planctomycetota*	69% *Thermogutta terrifontis*	41% *Thermotoga maritima* (3AMC)
5	*Dictyoglomota*	63% *Dictyoglomus thermophilum*	41% *Bacillus licheniformis* (4YZP)
5	*Dictyoglomota*	73% *Dictyoglomus thermophilum*	59% *Thermotoga maritima* (3AMC)
5	*Chloroflexota*	86% *Roseiflexus castenholzii*	22% *Bacteroides cellulosilyticus* (5JVK)
5	*Chloroflexota*	90% *Roseiflexus castenholzii*	29% *Acetivibrio thermocellus* (1CEC)
5	*Chloroflexota*	94% *Roseiflexus castenholzii*	25% *Bacteroides cellulosilyticus* (5JVK)
5	*Armatimonadota*	40% *Acaryochloris m*arina	28% *Bacteroides cellulosilyticus* (5JVK)
44	*Armatimonadota*	64% *Arenimonas oryziterrae*	69% uncultured bacterium (3FW6)
5	*Verrucomicrobiota*	72% *Limisphaera ngatamarikiensis*	40% *Thermotoga maritima* (3AMC)
5	*Verrucomicrobiota*	69% *Limisphaera ngatamarikiensis*	29% *Acetivibrio thermocellus* 1CEC)
5	*Verrucomicrobiota*	66% Limisphaera ngatamarikiensis	nil
5	*Verrucomicrobiota*	53% *Fontisphaera persica*	48% *Thermogutta terrifontis* (8AG9)
5	*Verrucomicrobiota*	75% *Limisphaera ngatamarikiensis*	44% *Thermotoga maritima* (7EC9)
5	*Myxococcota*	50% *Ktedonobacter* sp. SOSP1-85	44% *Acetivibrio thermocellus* (1CEC)
5	Myxococcota	34% *Occallatibacter savannae*	31% *Saccharophagus degradans* (5A8N)
**Xylanase**			
10	*Thermotogota*	55% *Petrotoga m*obilis	65% *Pseudothermotoga thermarum* (7NL2)
10	*Dictyoglomota*	73% *Dictyoglomus thermophilum*	51% *Geobacillus stearothermophilus* (1N82)
10	*Dictyoglomota*	77% *Dictyoglomus thermophilum*	29% *Paenibacillus b*arcinonensis (4XUO)
10	*Dictyoglomota*	72% *Dictyoglomus thermophilum*	54% *Thermotoga maritima* (1I82)
10	*Dictyoglomota*	73% *Dictyoglomus thermophilum*	51% *Geobacillus stearothermophilus* (2Q8X)
10	*Dictyoglomota*	77% *Dictyoglomus thermophilum*	43% *Caldicellulosiruptor acetigenus* (7NWN)
10	*Dictyoglomota*	48% *Dictyoglomus thermophilum*	28% *Paenibacillus barcinonensis* (4XUO)
10	*Dictyoglomota*	73% *Dictyoglomus thermophilum*	40% *Caldicellulosiruptor a*cetigenus (7NWN)
10	*Dictyoglomota*	73% *Dictyoglomus thermophilum*	51% *Geobacillus stearothermophilus* (1N82)
10	Armatimonadota	53% *Meiothermus h*ypogaeus	37% *Bacteroides thetaiotaomicron* (5XXL)
10	Verrucomicrobiota	65% *Limisphaera ngatamarikiensis*	33% *Acetivibrio clariflavus* (8B73)
10	Verrucomicrobiota	60% *Verrucomicrobium spinosum*	38% *Cellvibrio m*ixtus (2CNC)
10	Deinococcota	100% *Meiothermus luteus*	49% *Thermotoga maritima* (1VBR)
β**-glucosidase**			
1	Nitrospirota	85% *Thermodesulfovibrio* sp.	40% *Pyrococcus horikoshii* (1VFF)
3	Thermoproteota	75% *Dictyoglomus thermophilum*	45% *Acetivibrio thermocellus* (7MS2)
3	Thermoproteota	60% *Dictyoglomus thermophilum*	37% *Pseudomonas aeruginosa* (6R5O)
1	Spirochaetota	55% *Meiothermus ruber*	42% *Acetivibrio thermocellus* (5OGZ)
1	Caldatribacteriota	63% *Atribacter laminatus*	55% *Halothermothrix orenii* (4PTV)
1	Caldatribacteriota	*63% Atribacter laminatus*	54% *Niallia circulans* (1QOX)
3	Caldatribacteriota	66% *Dictyoglomus thermophilum*	37% *Bacteroides thetaiotaomicron* (5XXL)
1	WOR-3	42% Candidatus *Velamenicoccus archaeovorus*	40% *Pyrococcus horikoshii* (1VFF)
1	*Dictyoglomota*	70% *Dictyoglomus thermophilum*	57% *Halothermothrix orenii* (4PTV)
1	*Dictyoglomota*	70% *Dictyoglomus thermophilum*	42% *Pyrococcus horikoshii* (1VFF)
3	*Dictyoglomota*	82% *Dictyoglomus thermophilum*	47% *Saccharopolyspora erythraea* (5M6G)
3	*Dictyoglomota*	82% *Dictyoglomus thermophilum*	66% *Acetivibrio thermocellus* (7MS2)
1	CSP1-3	56% *Thermoflexus hugenholtzii*	41% *Pyrococcus horikoshii* (1VFF)
3	*Chloroflexota*	90% *Roseiflexus castenholzii*	43% *Kluyveromyces marxianus* (3AC0)
1	*Chloroflexota*	95% *Roseiflexus castenholzii*	40% *Pyrococcus horikoshii* (1VFF)
1	*Myxococcota*	54% *Atopomonas h*ussainii	38% *Pyrococcus horikoshii* (1VFF)
1	*Patescibacteria*	44% *Dictyoglomus thermophilum*	34% *Pyrococcus* horikoshii (1VFF)
3	*Deinococcota*	99% *Meiothermus* sp.	53% *Acetivibrio thermocellus* (7MS2)
1	*Deinococcota*	90% *Meiothermus luteus*	53% *Halothermothrix orenii* (4PTV)
1	*Deinococcota*	92% *Meiothermus luteus*	48% *Thermotoga maritima* (1OD0)
β**-xylosidase**			
3	*Thermoproteota*	71% *Dictyoglomus thermophilum*	54% *Thermotoga maritima* (7ZB3)
39	*Planctomycetota*	35% *Thermogutta terrifontis*	23% *Bacteroides c*ellulosilyticus (5JVK)
3	*Ratteibacteria*	54% *Caldanaerobius fijiensis*	50% *Thermotoga maritima* (7ZB3)
3	*Chloroflexota*	90% *Roseiflexus castenholzii*	53% *Thermotoga maritima* (7ZB3)
43	*Verrucomicrobiota*	70% *Fontisphaera persica*	63% *Bacteroides eggerthii* (6MLY)
43	*Deinococcota*	85% *Meiothermus ruber*	62% *Halalkalibacterium halodurans* (1YRZ)

## Discussion

### Sequencing strategies and bioinformatic integration

In early 2022, Oxford Nanopore launched an early access ligation sequencing kit (Q20+, K12 chemistry) with over 99% raw read accuracy. Nanopore long reads have two advantages: a larger N50 than Illumina-MAGs, resulting in less fragmented contigs, and longer assembled DNA. However, for our current experiment, the data clearly indicate that one single Nanopore flow cell on MinION is not sufficient to generate a high number of medium- to high-quality MAGs. Despite our intention to rerun the frozen extracted genome on another flow cell, unfortunately, Oxford Nanopore has discontinued the R10.4 temporary version. However, it is worth noting that they now offer the R10.4.1 version. We decided not to purchase and try the latter version due to inconsistencies in the experimental setup. While we have not performed it, we would likely need at least two flow cells on MinION for each sample if aiming for a higher total number of constructed MAGs based on Nanopore alone.

In the past, hybrid assembly of Illumina short-reads and long-reads from other platforms is one of the common practices for various applications such as pure genomes, mock communities, and real environmental microbiomes [41, 42]. Recent advances in Nanopore (R10.4.1 and onward) and PacBio HiFi reads have challenged the necessity of hybrid assembly. Some reports have demonstrated that long-reads alone can be sufficient, provided that the sequencing depth is adequate [11, 31]. At the time of writing, Kato et al. [11] is the only team that demonstrated the use of PacBio HiFi long reads for a Japanese hot spring sample. Although there is a scarcity of publications that specifically employ long-read techniques on hot spring biofilms, an ongoing inquiry revolves around whether hybrid assembly can adeptly leverage the advantages of Illumina deep sequencing data and Nanopore long reads within this domain. This query arises due to the lack of instances involving hot spring biofilms, and this is one of the reasons we adopted the current hybrid sequencing and assembly approach in building MAGs.

In general, our findings suggest that a hybrid assembly of short- and long reads is superior to assemblies based solely on short reads (Table 1). We explored hybrid assembly using MEGAHIT, a program that is less computationally intensive but less commonly employed for Illumina-Nanopore hybrid assembly. Conversely, HybridSPAdes, known for its wide application in hybrid assembly, is more computationally demanding. Our study suggests HybridSPAdes generally outperforms MEGAHIT. Notably, MEGAHIT hybrid-assembled MAGs, chosen by dRep, have a high count and certain MEGAHIT-assembled MAGs are much better in overall quality (Table 2). Thus, researchers planning hybrid assemblies should consider multiple assemblers rather than relying on a single approach.

Our data demonstrate that the amalgamation of short and long reads has substantially improved the overall quality metrics compared to Illumina-MAGs or Nanopore-MAGs alone. Despite this improvement, there are a few drawbacks, namely the high cumulative sequencing cost and lengthy computation time. Additionally, new Nanopore users may face challenges due to the unpredictability of total output reads resulting from poor sample pipetting into the flow cell. Researchers working with hot spring biofilms may want to consider HiFi long reads, especially on the PacBio system, as it may overcome the limitations stated above. However, readers should be aware that the quantity and quality of the extracted genome from biofilm may pose a challenge that needs to be overcome.

### Microbial assemblages in green and brown biofilms

The green biofilm, abundant in *Cyanobacteria*, exhibits limited diversity. Thermophilic cyanobacterium *Thermosynechococcus* (*Cyanophyceae*) was prevalent in various regions like Singapore and Taiwan hot springs [43, 44]. Surprisingly, *Thermosynechococcus* was notably absent in the SKY hot spring based on amplicon and MAG datasets, likely influenced by physicochemical factors in the water. Among the five *Cyanobacteria* MAGs within the green biofilm, four were likely filamentous, attaching to plant litter using EPS and non-flagellar appendages, aiding surface adherence. *Chloroflexota*, many of which are also likely filamentous [33], contribute to the green biofilm matrix too. Cryo-electron tomography of thermophilic *Roseiflexus castenholzii* and *C. aggregans* (both *Chloroflexota*) revealed long pili anchored near septa in multicellular filaments, akin to *Cyanobacteria* [45]. *Cyanobacteria* typically have type IV pilin (PilA), while *Chloroflexota* like *C. aggregans* employ distinct Tad or Flp pili [45]. The exact mechanism of Tad pilus-mediated adherence is not fully understood, but it is believed that these pili facilitate the attachment of bacteria to specific molecules on surfaces.

Green biofilm exhibits a notably loose structure and lacks the ability to form a compact matrix when manipulated with forceps. It did not exhibit the expected cohesion of a microbial mat and was instead found to lack a slimy texture usually associated with extracellular polymeric substances. The loosely structured nature of the green biofilm nonetheless provided a favourable environment for the colonisation of various types of microbiota. In a reciprocal relationship, the *Cyanobacteria* in the green biofilm may compensate for their lack of enzymes capable of decomposing plant litter. During periods of abundant sunlight and lower water temperatures, *Cyanobacteria* can generate their own food through photosynthesis. However, under conditions of limited sunlight and elevated water temperatures, a different strategy emerges. The loose biofilm structure seems to facilitate a symbiotic relationship, allowing *Cyanobacteria* to benefit from short sugars produced by extracellular hydrolases secreted by neighbouring microbiota. Nevertheless, this potential symbiosis requires further experimentation.

Distinct from the green biofilm, the brown biofilm exhibited a different microbiome profile. Based on the previous 16S rRNA bacterial V3–V4 amplicon data, approximately 50% of the total ASVs in the brown biofilm were related to the phylum *Chloroflexota*, while the remaining ASVs were primarily associated with *Thermotoga*, *Bacteroidota*, *Acidobacteriota*, and *Armatimonadota*. We initially expected that the diversity of *Chloroflexota* in green and brown biofilms would be somewhat similar. It is possible, however, that only four MAGs are shared between the two biofilm types, namely those related to the genera *Thermoflexus*, *Roseiflexus*, NAK19 (order *Anaerolineales*), and DRWP01 (order *Thermoflexales*). According to the data from the constructed MAGs, the brown biofilm is rich in the genera *Thermoflexus* and *Thermomicrobium*, as well as several novel taxa belonging to the phylum *Chloroflexota*, whereas the green biofilm is deficient in these genera (Fig. 4). In general, the *Chloroflexota* phylum can produce energy through both photoheterotrophy and chemoheterotrophy. Certain known members of *Chloroflexota* have been proposed to be thiotrophs, also known as chemolithotrophs that derive their energy by oxidising inorganic compounds, particularly those rich in sulfur, such as sulfur compounds, sulfides, or elemental sulfur [47]. With recorded temperatures reaching up to 77 °C at the sampling site and an average of 74 °C for the brown biofilm itself, the majority of brown biofilm microbiota shall employ various energy acquisition strategies, which include chemoheterotrophic, chemoorganotrophic, chemoautotrophic, or other mechanisms.

The presence of *Armatimonadota* in green and brown biofilm raises questions, given its limited type strains studies, and its potential for biofilm formation remains uncertain. However, a few of the detected MAGs exhibit genes associated with exopolysaccharide biosynthesis proteins, polysaccharide modifying proteins, and sequences related to pilus formation. Further investigation is warranted to elucidate the biofilm-forming potential of *Armatimonadota*-related cultured strain when they become available in the future. Based on limited understanding, *Armatimonadota* perform chemolithotrophy, oxidising inorganics such as hydrogen, sulfur, or iron, sometimes coupled it with autotrophic acetogenesis [11, 46]. Researchers posit that this phylum is capable of functioning as chemoorganoheterotrophs and contributes to biogeochemical cycling [8, 11].

### CAZymes in SKY hot spring biofilms: unveiling potential for lignocellulosic degradation

Starch-related industries seek thermostable enzymes for enhanced enzymatic processes. Numerous thermostable CAZymes and thermophiles have been thoroughly studied [51–61]. The variety of thermophiles and thermozymes in the SKY hot spring was more complex than that suggested from pure culture isolation or mixed culture in laboratory setups, or compost with elevated temperature [48–50].

The phylum *Armatimonadetes*, formerly known as candidate division OP10 (where OP refers to Obsidian Pool at the Yellowstone National Park), is speculated to be a plant biomass degrader. Currently, the *Armatimonadetes* phylum holds only a few cultured taxa, including mesophilic strains (*Armatimonas rosea*, *Capsulimonas corticalis*, and *Fimbriimonas ginsengisoli*), and the thermophilic *Chthonomonas calidirosea*. In a genome analysis of *C. calidirosea*, the sole culturable thermophilic *Armatimonadetes* found so far, researchers noted 65 GH enzymes within the bacterium [62]. However, that study found that pure culture of *C. calidirosea* was unable to hydrolyse linear polysaccharides such as cotton, Avicel, lignocellulosic pulp preparations, and specifically cellulose. However, the MAGs constructed from our study indicated the presence of cellulases (Table 3).

The scientific community should not only focus on the *Armatimonadetes* phylum but also turn attention to *Chloroflexota*. MAGs associated with *Chloroflexota* demonstrate a diverse range of amylolytic enzymes and cellulose-degrading enzymes, highlighting the importance of exploring the enzymatic potential within this phylum for various biotechnological applications. Upon closer analysis, it becomes evident that the protein sequences of the CAZymes in *Armatimonadetes* and *Chloroflexota* exhibit low similarity with other proteins, warranting further in-depth investigations of these candidates in future studies. We have analysed some CAZyme sequences from *Armatimonadetes* and *Chloroflexota*, predicted their overall structures using AlphaFold, and examined the protein domain architectures. Our findings suggest that these protein sequences are authentic CAZymes, albeit their activities require validation in subsequent work.

## Conclusion

In this study, we conducted a comprehensive analysis of microbial community composition, taxonomy, adaptation, and metabolic potential within the green and brown biofilms of the SKY hot spring. The green biofilm was primarily composed of *Cyanobacteria*, alongside other phyla like *Bacteroidota*, *Chloroflexota*, and *Acidobacteriota*. Unlike typical firm and slimy biofilms, the green biofilm exhibited a loose structure, promoting diverse microbiota colonisation, ecological diversity, and potential symbiotic interactions within the matrix. In contrast, the brown biofilm, thriving in higher temperatures, showcased a more diverse composition, encompassing archaea as well as a variety of bacterial phyla. Heat shock proteins were prevalent in both biofilm types, underscoring their pivotal role in maintaining protein stability and countering heat stress-induced protein denaturation and proteotoxicity. Both biofilms exhibited various CAZymes, signifying the cooperative efforts of diverse microbial taxa in converting plant lignocellulosic biomass. Noteworthy were *Armatimonadota* and *Chloroflexota* MAGs that showcased versatility in carbohydrate metabolism, possessing an array of hydrolases targeting diverse carbohydrates. Current commercial enzyme cocktails for lignocellulosic saccharification are typically sourced from a single type of mesophilic fungal strains, and these cocktails contain almost the complete range of necessary enzymes. However, our study has revealed that in natural geothermally heated environments where plant litter decomposition occurs, finding a single prokaryotic thermophile with all the required industrial enzymes may not be feasible because thermophiles and their enzymes often collaborate synergistically.

### Supplementary Information

**Additional file 1: Table S1.** Taxonomy of dereplicated MAGs

**Additional file 1: Table S2.** Protein fasta sequences of CAZymes used in Table 3.

## References

[1] Gallo G, Puopolo R, Carbonaro M, Maresca E, Fiorentino G. Extremophiles, a nifty tool to face environmental pollution: From exploitation of metabolism to genome engineering. Int J Environ Res Public Health. 2021;18. 10.3390/ijerph18105228 PMC8157027 34069056

[2] Reichart NJ, Bowers RM, Woyke T (2021). High potential for biomass-degrading enzymes revealed by hot spring metagenomics. Front Microbiol.

[3] Luo ZH, Narsing Rao MP, Chen H, Hua ZS, Li Q, Hedlund BP (2021). Genomic insights of “candidatus nitrosocaldaceae” based on nine new metagenome-assembled genomes, including “Candidatus Nitrosothermus” Gen Nov. and two new species of “candidatus nitrosocaldus”. Front Microbiol.

[4] Marín-Paredes R, Tapia-Torres Y, Martínez-Romero E, Quesada M, Servín-Garcidueñas LE (2021). Metagenome assembly and metagenome-assembled genome of “ candidatus aramenus sulfurataquae” from thermal sediments from the Los Azufres volcanic complex. Microbiol Resour Announc.

[5] Nagar S, Talwar C, Bharti M, Yadav S, Siwach S, Negi RK (2021). Metagenome-assembled genomes recovered from the datasets of a high-altitude Himalayan hot spring Khirganga, Himachal Pradesh, India. Data Brief.

[6] Reichart NJ, Bowers RM, Woyke T, Hatzenpichler R (2022). Metagenomes and metagenome-assembled genomes from substrate-amended hot spring sediment incubations from Yellowstone National Park. Microbiol Resour Announc.

[7] Das S, Nabi I, Mingma N, Sherpa T, Kumar S, Sharma P (2023). Baseline metagenome-assembled genome (MAG) data of Sikkim hot springs from Indian Himalayan geothermal belt (IHGB) showcasing its potential CAZymes, and sulfur-nitrogen metabolic activity. World J Microbiol Biotechnol.

[8] Allioux M, Yvenou S, Merkel A, Cozannet M, Aubé J, Pommellec J (2022). A metagenomic insight into the microbiomes of geothermal springs in the Subantarctic Kerguelen Islands. Sci Rep.

[9] Zhang Z, Liu T, Li X, Ye Q, Bangash HI, Zheng J (2023). Metagenome-assembled genomes reveal carbohydrate degradation and element metabolism of microorganisms inhabiting Tengchong hot springs, China. Environ Res.

[10] Bowers RM, Kyrpides NC, Stepanauskas R, Harmon-Smith M, Doud D, Reddy TBK (2017). Minimum information about a single amplified genome (MISAG) and a metagenome-assembled genome (MIMAG) of bacteria and archaea. Nat Biotechnol.

[11] Kato S, Masuda S, Shibata A, Shirasu K, Ohkuma M (2022). Insights into ecological roles of uncultivated bacteria in Katase hot spring sediment from long-read metagenomics. Front Microbiol.

[12] Zhang Y, Liu T, Li MM, Hua ZS, Evans P, Qu Y (2022). Hot spring distribution and survival mechanisms of thermophilic comammox Nitrospira. ISME J.

[13] Levy-Booth DJ, Hashimi A, Roccor R, Liu LY, Renneckar S, Eltis LD, et al. Genomics and metatranscriptomics of biogeochemical cycling and degradation of lignin-derived aromatic compounds in thermal swamp sediment. ISME J. 2021;15. 10.1038/s41396-020-00820-x PMC8027834 33139871

[14] Liew KJ, Liang CH, Lau YT, Yaakop AS, Chan K-G, Shahar S (2022). Thermophiles and carbohydrate-active enzymes (CAZymes) in biofilm microbial consortia that decompose lignocellulosic plant litters at high temperatures. Sci Rep.

[15] Vishnivetskaya TA, Hamilton-Brehm SD, Podar M, Mosher JJ, Palumbo AV, Phelps TJ (2015). Community analysis of plant biomass-degrading microorganisms from Obsidian Pool, Yellowstone National Park. Microb Ecol.

[16] Dixit S, Gaur M, Subudhi E, Sahoo RK (2021). Bacterial diversity and CAZyme potential revealed in Pandanus rich thermal spring cluster of India: a non-cultivable 16S rRNA sequencing approach. Front Microbiol.

[17] Dixit S, Sahoo K, Gaur M, Sahoo RK, Dey S, Gupta VK (2023). A meta-omics approach to explore the biofuel-producing enzyme potential from extreme environmental conditions. Renew Sustain Energy Rev.

[18] Chan CS, Chan KG, Tay YL, Chua YH, Goh KM (2015). Diversity of thermophiles in a Malaysian hot spring determined using 16S rRNA and shotgun metagenome sequencing. Front Microbiol.

[19] Lee LS, Goh KM, Chan CS, Annie Tan GY, Yin WF, Chong CS (2018). Microbial diversity of thermophiles with biomass deconstruction potential in a foliage-rich hot spring. Microbiologyopen.

[20] Martin M (2011). Cutadapt removes adapter sequences from high-throughput sequencing reads. EMBnet J.

[21] Li D, Liu CM, Luo R, Sadakane K, Lam TW (2015). MEGAHIT: an ultra-fast single-node solution for large and complex metagenomics assembly via succinct de Bruijn graph. Bioinformatics.

[22] Uritskiy GV, DiRuggiero J, Taylor J (2018). MetaWRAP—a flexible pipeline for genome-resolved metagenomic data analysis. Microbiome.

[23] Kang DD, Li F, Kirton E, Thomas A, Egan R, An H (2019). MetaBAT 2: an adaptive binning algorithm for robust and efficient genome reconstruction from metagenome assemblies. PeerJ.

[24] Alneberg J, Bjarnason BS, De Bruijn I, Schirmer M, Quick J, Ijaz UZ (2014). Binning metagenomic contigs by coverage and composition. Nat Methods.

[25] Wu YW, Simmons BA, Singer SW (2016). MaxBin 2.0: an automated binning algorithm to recover genomes from multiple metagenomic datasets. Bioinformatics.

[26] Parks DH, Imelfort M, Skennerton CT, Hugenholtz P, Tyson GW (2015). CheckM: assessing the quality of microbial genomes recovered from isolates, single cells, and metagenomes. Genome Res.

[27] Olm MR, Brown CT, Brooks B, Banfield JF (2017). DRep: a tool for fast and accurate genomic comparisons that enables improved genome recovery from metagenomes through de-replication. ISME J.

[28] Parks DH, Chuvochina M, Rinke C, Mussig AJ, Chaumeil PA, Hugenholtz P (2022). GTDB: an ongoing census of bacterial and archaeal diversity through a phylogenetically consistent, rank normalized and complete genome-based taxonomy. Nucleic Acids Res.

[29] Nagarajan NS, Arunraj SP, Sinha D, Babu V, Rajan V (2012). HSPIR : a manually annotated heat shock protein information resource. Bioinformatics.

[30] Huang L, Zhang H, Wu P, Entwistle S, Li X, Yohe T (2018). DbCAN-seq: a database of carbohydrate-active enzyme (CAZyme) sequence and annotation. Nucleic Acids Res.

[31] Sereika M, Kirkegaard RH, Karst SM, Michaelsen TY, Sørensen EA, Wollenberg RD (2022). Oxford Nanopore R1.04 long-read sequencing enables the generation of near-finished bacterial genomes from pure cultures and metagenomes without short-read or reference polishing. Nat Methods.

[32] Korelusova J, Kastovsky J, Komarek J (2009). Heterogeneity of the cyanobacterial genus synechocystis and description of a new genus. Geminocystis J Phycol.

[33] Kawai S, Martinez JN, Lichtenberg M, Trampe E, Kühl M, Tank M (2021). In-situ metatranscriptomic analyses reveal the metabolic flexibility of the thermophilic anoxygenic photosynthetic bacterium chloroflexus aggregans in a hot spring cyanobacteria-dominated microbial mat. Microorganisms.

[34] Garcia Costas AM, Liu Z, Tomsho LP, Schuster SC, Ward DM, Bryant DA (2012). Complete genome of Candidatus Chloracidobacterium thermophilum, a chlorophyll-based photoheterotroph belonging to the phylum Acidobacteria. Environ Microbiol.

[35] Balbay MG, Shlafstein MD, Cockell C, Cady SL, Prescott RD, Lim DSS, et al. Metabolic versatility of Caldarchaeales from geothermal features of Hawai’i and Chile as revealed by five metagenome-assembled genomes. Front Microbiol. 2023;14. 10.3389/fmicb.2023.1216591 PMC10547907 37799600

[36] Schuergers N, Wilde A (2015). Appendages of the cyanobacterial cell. Life.

[37] Rossi F, De Philippis R. Exocellular polysaccharides in microalgae and cyanobacteria: chemical features, role and enzymes and genes involved in their biosynthesis. In: The physiology of microalgae. 2016.

[38] Malygina A, Balkin A, Polyakova E, Stefanov S, Potekhin A, Gogoleva N (2023). Taxonomic diversity of the microbial biofilms collected along the thermal streams on Kunashir Island. Ecologies.

[39] Pysz MA, Conners SB, Montero CI, Shockley KR, Johnson MR, Ward DE (2004). Transcriptional analysis of biofilm formation processes in the anaerobic, Hyperthermophilic Bacterium Thermotoga maritima. Appl Environ Microbiol.

[40] Laksanalamai P, Robb FT (2004). Small heat shock proteins from extremophiles: a review. Extremophiles.

[41] Xia Y, Li X, Wu Z, Nie C, Cheng Z, Sun Y (2023). Strategies and tools in illumina and Nanopore-integrated metagenomic analysis of microbiome data. iMeta..

[42] Chen Z, Erickson DL, Meng J (2020). Benchmarking long-read assemblers for genomic analyses of bacterial pathogens using oxford nanopore sequencing. Int J Mol Sci.

[43] George C, Lim CXQ, Tong Y, Pointing SB (2023). Community structure of thermophilic photosynthetic microbial mats and flocs at Sembawang Hot Spring, Singapore. Front Microbiol.

[44] Cheng YI, Lin YC, Leu JY, Kuo CH, Chu HA (2022). Comparative analysis reveals distinctive genomic features of Taiwan hot-spring cyanobacterium Thermosynechococcus sp. TA-1. Front Microbiol.

[45] Gaisin VA, Kooger R, Grouzdev DS, Gorlenko VM, Pilhofer M (2020). Cryo-electron tomography reveals the complex ultrastructural organization of multicellular filamentous chloroflexota (Chloroflexi) bacteria. Front Microbiol.

[46] Carlton JD, Langwig MV, Gong X, Aguilar-Pine EJ, Vázquez-Rosas-Landa M, Seitz KW (2023). Expansion of Armatimonadota through marine sediment sequencing describes two classes with unique ecological roles. ISME Commun.

[47] Kostešić E, Mitrović M, Kajan K, Marković T, Hausmann B, Orlić S, et al. Microbial diversity and activity of biofilms from geothermal springs in Croatia. Microb Ecol. 2023; (in press). 10.1007/s00248-023-02239-1 PMC10640420 37209180

[48] Iacono R, Strazzulli A, Giglio R, Bitetti F, Cobucci-Ponzano B, Moracci M (2022). Valorization of biomasses from energy crops for the discovery of novel thermophilic glycoside hydrolases through metagenomic analysis. Int J Mol Sci.

[49] Santos-Pereira C, Sousa J, Costa ÂMA, Santos AO, Rito T, Soares P (2023). Functional and sequence-based metagenomics to uncover carbohydrate-degrading enzymes from composting samples. Appl Microbiol Biotechnol.

[50] Wang S, Meng Q, Zhu Q, Niu Q, Yan H, Li K (2021). Efficient decomposition of lignocellulose and improved composting performances driven by thermally activated persulfate based on metagenomics analysis. Sci Total Environ.

[51] Gomez Del Pulgar EM, Saadeddin A (2014). The cellulolytic system of *Thermobifida fusca*. Crit Rev Microbiol.

[52] Ajeje SB, Hu Y, Song G, Peter SB, Afful RG, Sun F (2021). Thermostable cellulases/xylanases from thermophilic and hyperthermophilic microorganisms: current perspective. Front Bioeng Biotechnol.

[53] Khaswal A, Chaturvedi N, Mishra SK, Kumar PR, Paul PK (2022). Current status and applications of genus *Geobacillus* in the production of industrially important products—a review. Folia Microbiol.

[54] Liu Y, Sun Y, Wang H, Tang L (2019). Characterization of a novel multi-domain xylanase from Clostridium clariflavum with application in hydrolysis of corn cobs. Biotechnol Lett.

[55] Berisio R, Barra G, Romano M, Squeglia F, Ruggiero A (2022). Structural and biochemical characterization of endo-β-1,4-glucanase from *Dictyoglomus thermophilum*, a hyperthermostable and halotolerant cellulase. Catalysts.

[56] Chan CS, Sin LL, Chan KG, Shamsir MS, Manan FA, Sani RK (2016). Characterization of a glucose-tolerant β-glucosidase from Anoxybacillus sp DT31. Biotechnol Biofuels.

[57] Li X, Xia J, Zhu X, Bilal M, Tan Z, Shi H (2019). Construction and characterization of bifunctional cellulases: *Caldicellulosiruptor*-sourced endoglucanase, CBM, and exoglucanase for efficient degradation of lignocellulose. Biochem Eng J.

[58] Kim SK, Russell J, Cha M, Himmel ME, Bomble YJ, Westpheling J (2021). Coexpression of a β-D-xylosidase from *Thermotoga maritima* and a family 10 xylanase from *Acidothermus cellulolyticus* significantly improves the xylan degradation activity of the *Caldicellulosiruptor bescii* exoproteome. Appl Environ Microbiol.

[59] Bing RG, Sulis DB, Wang JP, Adams MWW, Kelly RM (2021). Thermophilic microbial deconstruction and conversion of natural and transgenic lignocellulose. Environ Microbiol Rep.

[60] Chettri D, Verma AK, Sarkar L, Verma AK (2021). Role of extremophiles and their extremozymes in biorefinery process of lignocellulose degradation. Extremophiles.

[61] Sahoo K, Sahoo RK, Gaur M, Subudhi E (2020). Cellulolytic thermophilic microorganisms in white biotechnology: a review. Folia Microbiol (Praha).

[62] Lee KCY, Morgan XC, Dunfield PF, Tamas I, McDonald IR, Stott MB (2014). Genomic analysis of Chthonomonas calidirosea, the first sequenced isolate of the phylum Armatimonadetes. ISME J.

